# Direct improvement of quality of life in colorectal cancer patients using a tailored pathway with quality of life diagnosis and therapy (DIQOL): study protocol for a randomised controlled trial

**DOI:** 10.1186/s13063-015-0972-y

**Published:** 2015-10-14

**Authors:** Monika Klinkhammer-Schalke, Patricia Lindberg, Michael Koller, Jeremy C. Wyatt, Ferdinand Hofstädter, Wilfried Lorenz, Brunhilde Steinger

**Affiliations:** Tumor Center Regensburg e.V., An-Institute of the University of Regensburg, Josef-Engert-Straße 9, 93053 Regensburg, Germany; Center for Clinical Trials, University Hospital Regensburg, Franz-Josef-Strauß-Allee 11, 93053 Regensburg, Germany; Leeds Institute of Health Sciences, University of Leeds, 101 Clarendon Road, Leeds, LS2 9LJ UK; Johannes Kepler Universität Linz, Medizinische Fakultät, Linz, Austria; Tumorzentrum Regensburg e.V., An-Institut der Universität Regensburg, Josef-Engert-Str. 9, 93053 Regensburg, Deutschland

**Keywords:** Quality of life, colorectal cancer, complex intervention, randomised controlled trial, study protocol

## Abstract

**Background:**

Medical treatment in patient-centred care in oncology is broadly managed and regulated in terms of guideline development, cancer centres, and quality assurance by cancer registries. In contrast to this quality management cycle (PDCA), there are no equal standards for patient-reported outcomes like quality of life (QoL). Therefore, the Tumour Centre Regensburg e.V., a population-based regional cancer registry covering a population of more than 2.2 million people in the Upper Palatinate and Lower Bavaria, Germany, designed and implemented a QoL pathway. In a complex intervention with QoL diagnosis and therapy (multidimensional therapeutic network), effectiveness for patients with breast cancer has been demonstrated. To provide local tailored QoL diagnosis and therapy to other cancer patients as well, external validity needs to be extended by adapting the QoL pathway to another tumour entity.

**Methods/Design:**

The QoL pathway will be tested for colorectal cancer patients in a pragmatic randomised controlled trial. Two hundred twenty primary colorectal cancer patients, surgically treated in one of four hospitals, will be included. QoL is measured in all patients 0, 3, 6, 12, and 18 months after surgery (European Organisation for Research and Treatment of Cancer (EORTC) QLQ-C30, QLQ-CR29). In the intervention group, QoL scores are transformed into a QoL profile. This is sent to the coordinating practitioner (general practitioner, internist, or oncologist) with an expert report including treatment recommendations for QoL deficits. The control group receives routine follow-up care attending the guideline recommendations for colorectal cancer without profile or expert report. At the primary endpoint (12 months), the rates of patients with diseased QoL in both groups are compared.

**Discussion:**

This randomised trial is the first complex intervention investigating the effectiveness of an intervention with QoL diagnosis and tailored QoL therapy in colorectal cancer patients. The results will show if this QoL pathway improves the patients’ QoL during follow-up care of their disease.

**Trial registration:**

ClinicalTrials.gov, NCT02321813 (registered December 2014).

## Background

There is a growing interest in using quality of life (QoL) data not only as a relevant endpoint in clinical trials on cancer patients, but also in routine practice in order to improve patients’ health during treatment [1–4].

Until now, several essentials for managing ‘diseased QoL’ have been missing. First of all, there is a need of guidance about how to define and use diseased QoL as an integral part of cancer treatment, for example, in patients with good prognostic stage (UICC 0 and I) but persistently low QoL in several dimensions requiring the doctor’s help [5]. Furthermore, therapies rarely have been implemented specifically to improve patients’ QoL. Additionally, there needs to be confidence in the evidenced-based value of such QoL therapies [6].

To achieve these aims, we designed, implemented, and evaluated an integrated QoL diagnosis and therapy pathway (QoL pathway) for breast cancer patients, guided by the UK Medical Research Council framework for developing and testing complex interventions [7]. In a previous randomised controlled trial it could be demonstrated in a routine setting that breast cancer patients show a benefit from tailored QoL diagnosis and therapy, having significantly better QoL 6 months after surgery compared to a control group treated with routine follow-up care [6].

In order to extend the external validity of our approach it is necessary to apply its principles to another tumour entity. Colorectal cancer was chosen for the following reasons: It is the second most common form of cancer in the Western world (69,000 new diagnosed patients/year in Germany), responsible for about 27,000 cases of death per year in Germany [8]. The demands of this cancer form on QoL diagnosis and therapy differ in some important aspects from those in breast cancer (for example, more important part of surgical procedure because of the more extended severe tumour extirpation and patients of both sexes, with higher age and more comorbidities).

Many studies have demonstrated that various components of the QoL of colorectal cancer patients are considerably impaired. QoL deficits are most dominant during the first months after surgery with some discomfort persisting for a longer time, for example, impaired body image or sexual problems [9]. One year after diagnosis, colorectal cancer patients still suffer fatigue and reduced emotional functioning compared to the general population [10]. Special aspects concerning this form of cancer are stoma-related complications. The presence of a stoma is associated with more psychological distress and reduced sexual functioning [11] while stoma reversal leads to a significant improvement in physical and social functioning and overall QoL [12]. Furthermore, it was shown that baseline QoL is a significant prognostic indicator for survival in advanced colorectal cancer, with better QoL being associated with a higher 1-year survival rate [13].

There is general agreement that QoL needs to be not only considered as an endpoint in clinical trials but also in routine health care of cancer patients. The mainstream of research has focused on developing a routine measurement of QoL in clinical practice and providing the results to clinicians in order to improve patient-physician communication [1, 2, 14, 15]. These studies, however, provide no clues how to deal with this information because specific implications for the treatment of QoL deficits never have been spelled out.

Therefore, we developed a QoL pathway, which connects a systematic QoL diagnosis with targeted treatment of QoL deficits in breast cancer patients. Because of the complexity of the healthcare system, the study was conducted as a complex intervention [7, 16] with theory building [17, 18], modelling [5], exploratory trial [19], and a definitive randomised controlled trial [6]. In the present randomised trial, effectiveness of the QoL pathway, modified for colorectal cancer patients, is to be tested.

### Objectives and hypothesis

The primary objective of the study is to improve QoL of colorectal cancer patients during follow-up care with systematic QoL diagnosis and targeted treatment. To achieve this aim, the following key issues are addressed:

1. Testing effectiveness of the QoL pathway with systematic diagnosis and targeted therapy of QoL in colorectal cancer patients during follow-up care.
2. Implementing the QoL system into routine medical care using a multifaceted strategy including educational outreach visits, local opinion leaders, and continuous medical education (CME) (in defined and modelled quality circles [5]).
3. Extension of regional network structures for QoL therapies by creating new quality circles and by expanding the already existing quality circles.
4. Extension of external validity by applying the QoL pathway in another tumour entity.
5. Replication of the findings as an essential part of complex interventions.

The following hypothesis will be investigated:

Colorectal cancer patients who receive systematic QoL diagnosis and targeted QoL therapy (intervention) have a significantly better QoL in the first postoperative year compared to patients who receive routine follow-up care (control).

## Methods/Design

The study is a two-armed, randomised, controlled, prospective, single-blind, pragmatic, clinical trial with an intervention group and a control group.

### Participants and recruitment

Four hospitals with certified colorectal cancer centres in Bavaria, Germany, accredited by the German Cancer Society (DKG), take part in patient recruitment: Krankenhaus Barmherzige Brüder, Department of Surgery, Regensburg; Caritas-Krankenhaus St. Josef, Department of Surgery, Regensburg; Klinikum Neumarkt, Department of Surgery, Neumarkt; Klinikum St. Elisabeth Straubing, Department of Surgery, Straubing. Before the beginning of the trial the number of patients fulfilling the inclusion criteria has been collected in the four hospitals for the year 2011 in order to see if enough eligible patients are available. There has been a pilot phase in the hospitals for the QoL pathway in order to implement patient recruitment and postsurgical QoL measurement into clinical routine. To achieve an adequate participant enrolment there is regular contact between the QoL Unit and the surgeons recruiting patients in the four hospitals. Moreover, the participating clinics are certified cancer centres that need enrolment of patients into studies for the certification process and thus should be highly motivated to include patients in the trial.

**Fig. 1:**
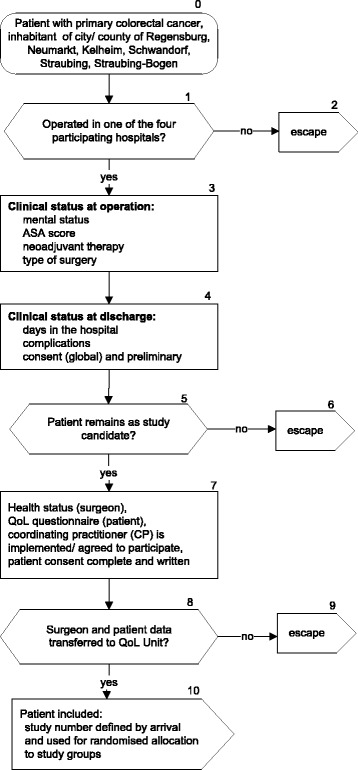
Selection of patients

Patients are included under broad inclusion criteria: (1) diagnosis of primary colorectal cancer and (2) surgery in one of the four participating hospitals. Exclusion criteria are as follows: (1) recruiting surgeon (QoL clinician) is not available (illness, holiday, other reasons); (2) patient is misclassified in the operation schedule (no primary operation or no colorectal tumour); (3) coordinating practitioner (physician, caring for the patient) chosen by the patient refuses trial participation; (4) patient is from outside the defined study region (Bavaria, Germany: urban county of Regensburg, rural county of Neumarkt, Straubing, Straubing-Bogen, Kelheim, Schwandorf); (5) age under 18 years; (6) pregnancy; (7) patient unable to fill out the QoL questionnaire for physical, psychological, or language reasons (including dementia); or (8) patient refuses to participate in the study. To insure high external validity [20] , there will be no exclusion of elderly patients or exclusion according to tumour stage or patient sex. Furthermore, no constraints will be made according to surgical procedure or ASA score (American Society of Anesthesiologists); however, these factors will be accounted for in data analyses. External validity is also kept high by population-based inclusion of patients of urban and rural areas and by implementing the QoL pathway into routine follow-up care. Moreover, data of the cancer registry of excluded patients and other hospitals in the study region can be analysed according to medical and demographic data of patients to test representativeness of our study population. The selection of study participants is shown in Fig. 1.

Coordinating practitioners (CPs) are defined as those physicians who care for the colorectal cancer patients after hospital stay. They are either general practitioners, or internists, or oncologists. CPs are eligible for the project if they give their informed consent to participate in the trial (CP is informed by the patient’s surgeon by phone about the study) and agree to be implemented by an educational outreach visit. Outreach visits are performed by two trained individuals of the QoL Unit who meet with the CP in his practice and introduce him to the concept of QoL, stressing the importance of considering QoL in medical care of colorectal cancer patients and who give the CP an overview about the procedure of the study. Outreach visits are structured by using fixed presentation slides for the implementation of each CP.

### Randomisation and blinding

Balanced randomisation [21] is used with random permuted blocks of 20 patients followed by a sequence of 11 blocks for 220 patients with a second, simple randomisation. Five digit random numbers from the RAND Corporation [22] are used for both steps. Based on this random list of digits an external co-worker, otherwise not involved in the trial, created 220 paper cards specifying the groups and inserted them in 1 to 220 serially numbered opaque, sealed envelopes. This stack of envelopes is kept in a locked safe at the QoL Unit at the Tumour Centre Regensburg and is accessible only by the two study coordinators.

Randomisation of individual patients is performed using the following described procedure. The study clinician in each of the four recruiting hospitals determines eligibility of the study patient. If all criteria are fulfilled and the patient has granted informed consent the study clinician sends the recruitment document to the QoL Unit. The study number 1 to 220 is determined by the exact date and time of fax entry. The CP will be informed by the QoL Unit about allocation of the individual patient either to the intervention or the control group.

The CP will be obliged not to share this knowledge with the patient. In order to control for bias, participants will be kept blind throughout the study about their allocated group (single blind design). At the end of the trial, the patients’ blindness about their allocated group will be checked.

At no point in the process will the recruiting clinician in the hospital access the sealed envelopes or be informed about the group allocation.

### Procedure

Written informed consent is obtained from each participant before they are included into the study. All participants (intervention and control group) repeatedly fill out a QoL questionnaire (EORTC QLQ-C30, QLQ-CR29 [23–25]) at the following times: 0 to 2 days before discharge from hospital and 3, 6, 12 and 18 months postoperatively. The first QoL measurement takes place in hospital and the further measurements (3, 6, 12, 18 months postoperatively) are carried out in the CP’s practice during follow-up. For each assessment time point participants receive a postal reminder. Guideline for colorectal cancer [26] recommends first follow-up at 6 months. Colorectal cancer patients suffer a lot of QoL deficits especially in the first months of treatment. Therefore, we decided also to measure QoL 3 months postoperatively. Feasibility of this additional measurement is expected to be good because most patients visit their CP regularly. This could be also observed in the pilot phase of the study.

Parallel to the QoL measurements, the patient’s CP provides additional information about the patient’s health status. All data are sent to the QoL Unit at the Tumour Centre Regensburg.

All patients are treated according to the follow-up regimen of S3-guideline for colorectal cancer [26]. Concomitant care is not prohibited but will be collected and considered in secondary data analysis.

**Fig. 2:**
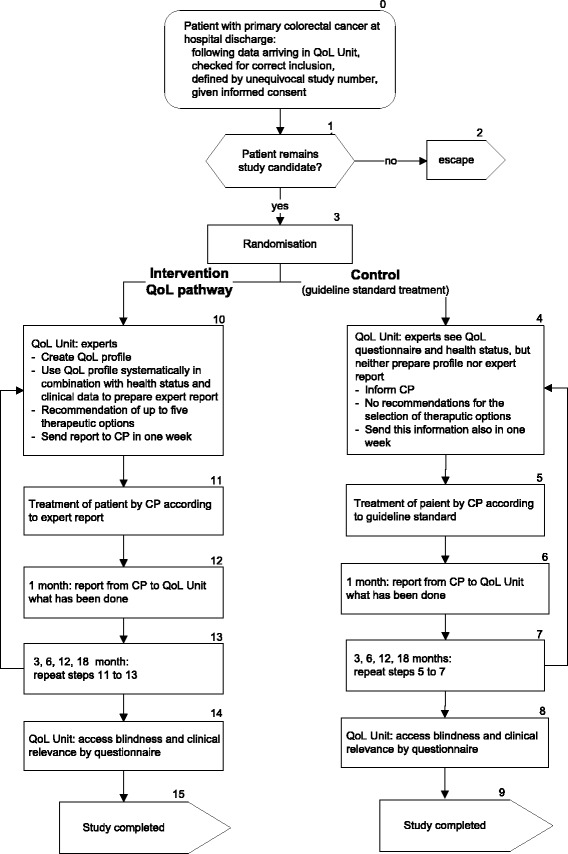
Trial profile: patients flow through each stage in two arms of the randomised controlled trial (CP, coordinating practitioner)

Differences between the treatment of intervention group and control group patients is shown in the following section. Figure 2 gives an overview of the study flow in the two arms of the randomised trial.

#### Intervention group

**Fig. 3:**
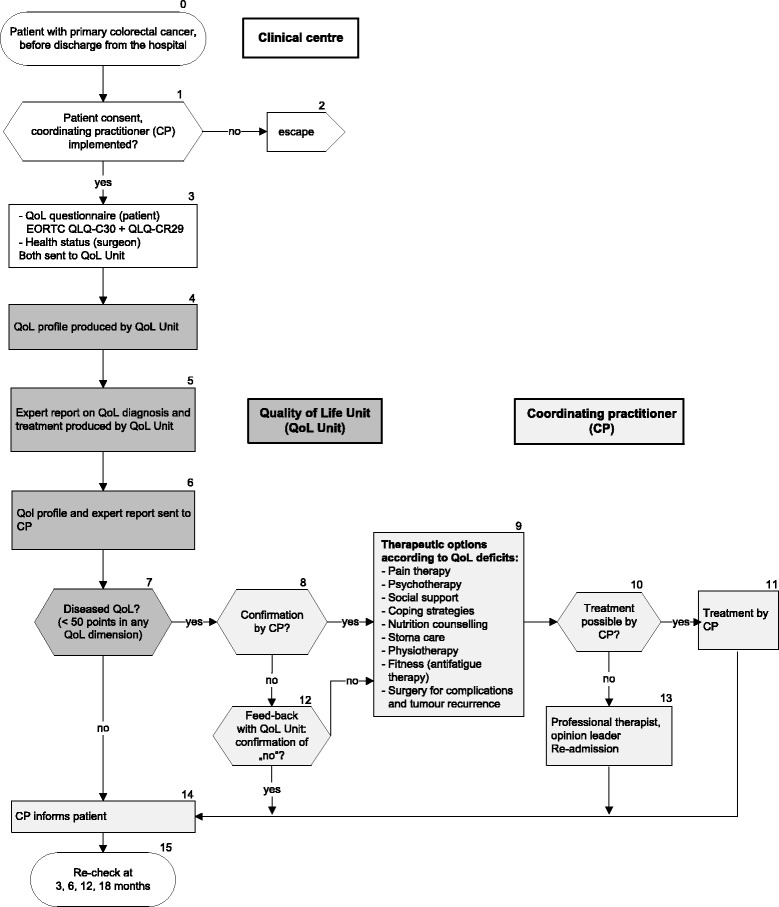
Patients in the intervention group (quality of life (QoL) pathway) following recruitment for the randomised trial. This pathway is considered for clinical routine

**Fig. 4:**
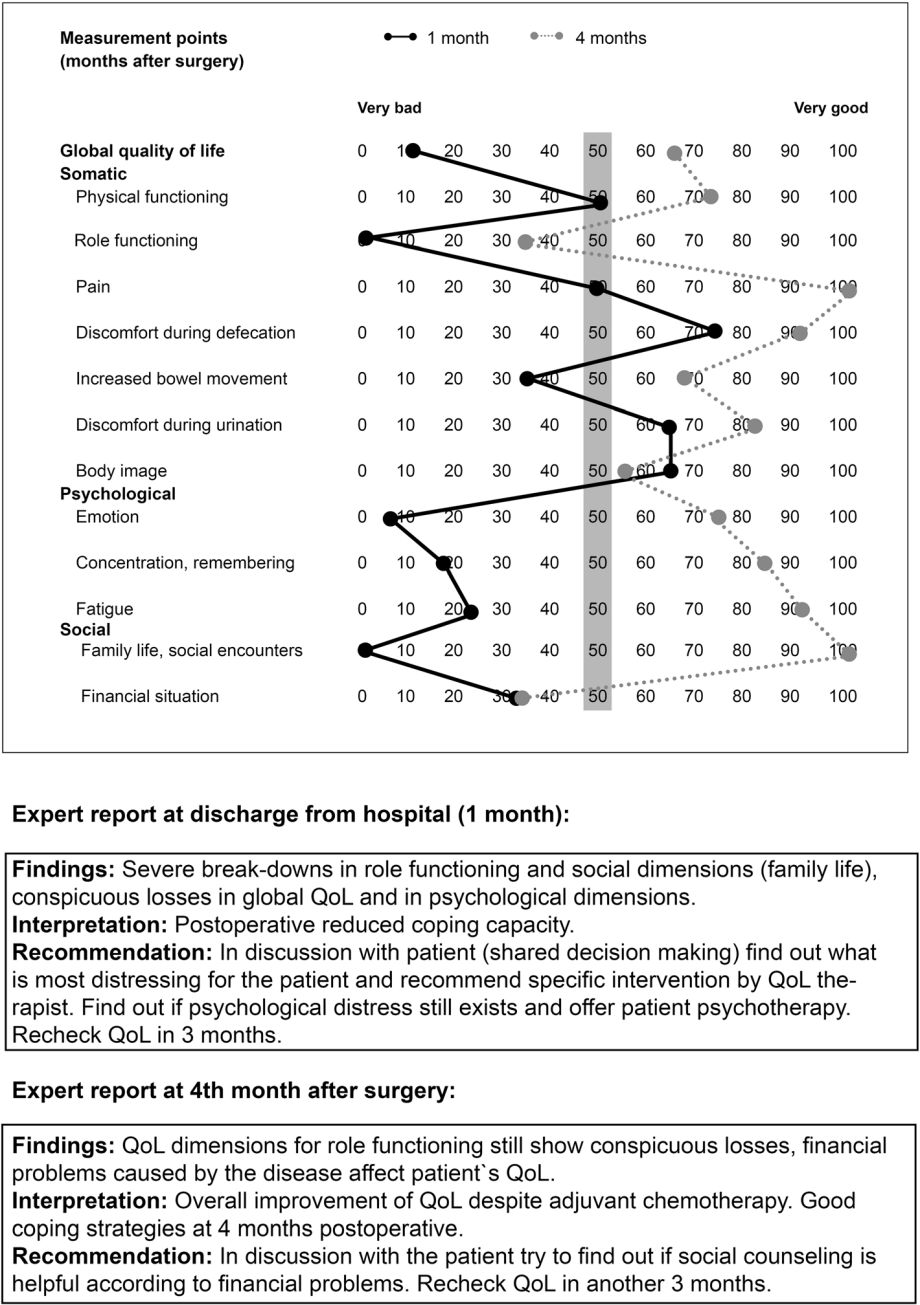
Quality of life (QoL) profile and expert reports of a colorectal cancer patient (pilot study) with QoL diagnosis and therapy. Male, with primary colon carcinoma, 56 years. Prognostic classification T3 N2a M1 (per) G3. Left hemicolectomy, subtotal pancreas resection, splenectomy. Adjuvant chemotherapy. *Grey bar* = Cut-off for diseased/healthy QoL (50 points)

The QoL pathway for intervention group patients is shown in Fig. 3. All intervention group patients receive, in addition to routine follow-up care [26], tailored QoL diagnosis and therapy during the first 18 months after surgery. For this purpose measurements of a QoL questionnaire (EORTC QLQ-C30, QLQ-CR29 [23-25]) are transformed into a QoL profile using a computerised visualisation programme [27]. The profile consists of scales of 0 to 100 (worst to optimal QoL) comprising global QoL and 12 different dimensions considering somatic (e.g. pain), psychological (e.g. emotional functioning), and social aspects (e.g. family life) (Fig. 4). The QoL profile also takes into account the specific discomfort colorectal cancer patients suffer, for example, disability during defecation, increased bowel movement, or discomfort during urination (EORTC QLQ-CR29). A cut-off score has been constructed, which defines scores < 50 points as ‘diseased QoL’.

The QoL profile and information about health status are interpreted by a group of experts in the QoL Unit (physicians and psychologist) who independently formulate a QoL diagnosis and recommendations for the treatment of diseased QoL deficits (<50 points). These are combined to a consensus report, which will be sent with the QoL profile (Fig. 4) to the CP caring for the patient. Four weeks after having received the expert report, the CP will be asked by phone by the QoL Unit, if any therapy has been performed in response to QoL diagnosis.

**Fig. 5:**
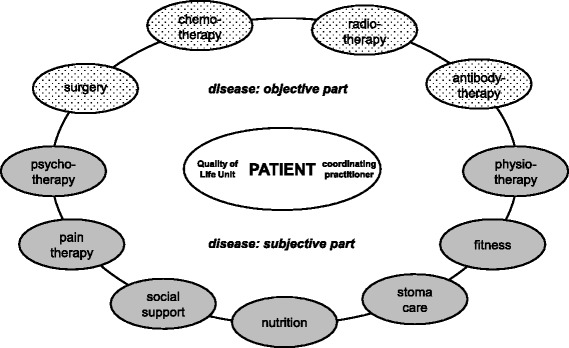
Traditional and hermeneutic therapeutic options for patients with colorectal cancer. An integrated model

Specific therapeutic options for the treatment of diseased QoL have been defined (Fig. 5): pain therapy, psychotherapy, social support, nutrition counselling, stoma care, physiotherapy, fitness. To provide continuous medical education, quality circles for each therapy option are necessary. As a result of the complex intervention in breast cancer patients [5, 6, 19], regional network structures for QoL therapies have been established and are available for the current study. To meet the special needs of colorectal cancer patients a new quality circle for nutrition and stoma care has been founded. CPs of intervention group patients receive addresses of the quality circle members to contact them easily for QoL therapy.

QoL of patients will be rechecked at 3, 6, 12, and 18 months postoperatively during follow-up in the practice of the CP with QoL diagnosis and therapy following the same procedure as described above.

#### Control group

In the control group patients’ QoL is also assessed before clinical discharge and at 3, 6, 12, and 18 months postoperatively during medical follow-up. In contrast to the intervention group, the CP of a control group patient is *not* informed about the results of patient’s QoL measurement (neither the QoL profile nor expert report are sent to him). Patients receive standard follow-up care according to the S3-guideline for colorectal cancer [26] (see Fig. 2).

### Measures

#### Quality of life (patient)

Quality of life is measured by using the validated QoL questionnaire of the European Organisation for Research and Treatment of Cancer (EORTC), consisting of a core module (EORTC QLQ-C30 [25, 28]) and a module for colorectal cancer patients (EORTC QLQ-CR29 [23, 24]). The questionnaire is a self-administration instrument with a multidimensional structure measuring QoL on a four-point Likert scale or rather on a seven-point Likert scale for the global dimension QoL. Satisfying internal consistency and good retest-reliability, as well as construct and clinical validity, have been demonstrated [24, 28, 29].

In every dimension scores are uniformly transferred to a scale from 0 (very bad QoL) to 100 (very good QoL). A cut-off score has been constructed, which defines scores < 50 points as diseased QoL. This score has been extensively discussed and accepted in the present form by consensus of a large group of clinicians [5, 6, 18].

For outcome measurement of the present randomised trial, 13 different scales have been selected according to the results of the previous randomised trial in breast cancer patients [6], the appraisal of relevance for QoL of colorectal cancer patients, and the availability of therapy options (global QoL, physical functioning, role functioning, pain, discomfort during defecation, increased bowel movement, discomfort during urination, body image, emotion, concentration/remembering, fatigue, family life/social encounters, and financial situation).

#### Health status (surgeon/CP)

The first measurement of health status is performed before discharge from the hospital. The surgeon gives information about the following demographic and medical characteristics of the patient: age, sex, marital status, children, occupation, ASA classification, tumour stage, date of surgery, surgical procedure, complications, comorbidities, (neo)adjuvant therapies, other current or planned therapies (for example, pain therapy, psychotherapy physiotherapy), CEA, preoperative symptoms, height, and weight. The type of surgery and ASA classification especially are expected to affect strongly colorectal cancer patients’ QoL [30]. These variables will be considered in data analysis, but neither specific surgical procedures nor higher ASA classifications will be excluded in order to achieve high external validity.

During follow-up the CP gives written information about health status at 3, 6, 12 and 18 months postoperatively regarding CEA, finished, current, or planned therapies, and complications. Additionally, the CP makes an assessment of the patient’s global QoL on a seven-point Likert scale.

#### Telephonic enquiries (CP)

Four weeks after the patient has filled out the QoL questionnaire, a member of the QoL Unit calls the CP by telephone and asks if anything has been done to improve the patient’s QoL. The reason for this is that in the intervention group CPs receive a QoL profile and an expert report with recommendations for improving their patients’ QoL. Therefore, whether anything has been done in response to the expert report is to be recorded by phone call. In order to ensure comparability of patients in both study arms, there are also telephonic enquiries for CPs of control group patients.

#### Patient satisfaction (patient)

After the patient has finished the last QoL measurement at 18 months postoperatively, a questionnaire will be sent to the patient’s home. The questionnaire includes quantitative and qualitative questions on patient satisfaction regarding (medical) care since the diagnosis of colorectal cancer and usefulness of the repeated QoL measurements. Additionally, the blindness of patients about their allocated group is checked.

### Ethics and monitoring

This study has received full ethical approval through the ethics committee of the University of Regensburg (internal reference number 12-101-0014). The trial is registered with ClinicalTrials.gov, trial registration number NCT02321813.

A central ethical question has to be answered before conducting a randomised trial: Is a new treatment denied to the control group from which those might benefit? In our study, control group patients are treated according to the S3-guideline for colorectal cancer (26) but do not receive additional targeted QoL therapy based on systematic diagnosis. Nevertheless, it can be expected that control group patients also benefit from study participation because their CP will also be trained in assessing QoL and therefore is expected to be sensitised for QoL deficits of his patient. It must also be considered that the new intervention or specific treatment of QoL might bear a risk for patients in the intervention group. Furthermore, emotional drain by filling out QoL questionnaires is conceivable. An insurance for patients’ safety was taken out to cover any possible harm of trial participants.

The randomised trial on breast cancer patients [6] did not show any evidence of possible harm elicited by the QoL pathway. For monitoring patient safety, patient’s CP is repeatedly contacted by phone about 1 month after the patient has filled out the QoL questionnaire (telephonic enquiries) and is asked whether he has recognised any negative effect on the patient. Serious adverse events, including death or admission to hospital, will be recorded, analysed, and reported to the local ethics committee.

### Primary and secondary outcomes

The first primary endpoint (1) will be the proportion of patients in both groups with diseased QoL (<50 points in at least one dimension) 12 months postoperatively. Twelve months were chosen because adjuvant therapies and stoma reversal are often not finished 6 months postoperatively.

The second primary endpoints (2), enhancing the credibility of the first primary endpoint, are the rates of patients with diseased QoL in each dimension of the profile 12 months after surgery.

The primary endpoints formulated above will be tested in the strict order (1), (2), that is, a statistical testing procedure with a priori ordered hypotheses is applied [31]. This binary primary endpoint was chosen for two reasons: (1) according to our conceptual groundwork [5, 19] 50 score points on a 0 to 100 QoL scale defines healthy versus diseased QoL, and (2) the same primary endpoint was used in our randomised trial on breast cancer patients and generated a significant difference according to our hypothesis [6].

### Sample size

The trial sample size was calculated by means of the first primary endpoint (lack of a QoL deficit in the QoL profile, that is, all scores are ≥ 50 score points). Based on the randomised controlled trial on breast cancer patients (6), we expect that 45 % of the patients in the intervention group report no QoL deficit 12 months after therapy, but only 25 % in the control group. Setting α at 5 % and power (1-β) at 80 %, 89 patients are needed in each group in order to detect the hypothesised difference when calculating the *Χ*^2^ test. To compensate for 20 % drop-outs (death or refusal) within the 12 months observation period, we will enrol 110 patients per group, and thus, the total sample size will be 220.

### Statistical analyses

Trial data are entered into a Microsoft ACCESS (version 2007) database, which is installed on a password-protected computer without physical connection to the internet. All data entered into the database are controlled by a second, independent member of the QoL Unit.

All variables will be presented using descriptive statistics (frequencies, percentages, means/standard deviations, medians/interquartile ranges, and various graphical representations). Point estimates will be accompanied by confidence intervals.

The design for the two primary endpoints is based on a method of fixed a priori ordered hypotheses [31]. Thus, the second primary endpoints will only be analysed, if the null hypothesis of the first primary endpoint can be rejected (*P* < 0.05). This design assures a global alpha of 0.05. The analyses of the primary endpoints (including both first and second primary endpoints) will be conducted using the *Χ*^2^ test (or Fisher’s exact test if the smallest expected cell value is < 5), setting the level of statistical significance at *P* < 0.05. It is not planned to use imputation to handle missing data in primary analysis.

Statistical tests regarding the secondary endpoints will also use the *P* < 0.05 significance threshold, but all secondary results will be interpreted in an exploratory manner.

Secondary analyses will start with an ANCOVA using the continuous QoL data as dependent variable and baseline QoL, comorbidity, sex, age and study site as covariates. Other secondary analyses will focus on exploratory subgroup analyses. These include the influence of different surgical procedures on QoL and the benefit these different subgroups gain from the QoL pathway. Furthermore, risk factors for diseased QoL (ASA, stoma, type of tumour (colon/rectum), age and sex) will be investigated. Tests of significance will include the Mann-Whitney *U* test, Kruskal Wallis test, *t*-test, and ANOVA or McNemar, depending on the number of groups, their independence/dependence, and the scaling of the dependent variable.

For qualitative data in the final questionnaire about patient satisfaction, a category system will be created and answers will be categorised by independent raters, so that frequencies of key categories can be quantitatively analysed by using descriptive statistics [32].

A detailed statistical analysis plan (SAP) will be written before all analyses commence.

## Discussion

In a complex intervention a clinical pathway with QoL diagnosis and QoL therapy has been developed, implemented, and the effectiveness has already been demonstrated for breast cancer patients. To provide the QoL pathway to other cancer patients as well, external validity needs to be extended by adapting the QoL pathway to another cancer type.

This randomised trial is the first complex intervention investigating the effectiveness of an intervention with regular measurement and treatment of QoL in colorectal cancer patients. The primary objective is to improve the QoL of the patients during follow-up care by systematic diagnosis and tailored therapy of diseased QoL. This regular screening of QoL combined with targeted therapy is supposed to support the prevention of chronic QoL deficits like fatigue or reduced emotional functioning.

Therefore, the treatment of cancer patients’ QoL needs to be just as standardised as medical therapy. This is a major strength of this study. In contrast to other interventional trials on QoL, the setting of this study is not limited to the inpatient process but encompasses especially ambulant routine follow-up care. Furthermore, the QoL pathway exceeds other approaches to improve QoL by not only measuring QoL and presenting results but also giving recommendations how to improve QoL, providing regional network structures for QoL therapy. Additionally, the trial is expected to have high external validity, for example, no exclusion of older patients, of specific tumour stages, or of patients with comorbidities. Therefore, the study has been carefully designed to reflect routine attending the recommendations in the S3-guideline. The four cancer centres responsible for patient recruitment are considered to be representative for institutions in which the QoL system is planned to be implemented in routine health care in the future. During the recruitment phase all colorectal cancer patients, surgically treated in the four hospitals, will be screened for study eligibility. We also expect a special motivation of CPs to participate in the trial because they are informed about the study by their patient’s surgeon and are implemented by an educational outreach visit. Moreover, thorough planning and a lot of preliminary work have been done in the course of the complex intervention including theory building [17, 18], modelling [5], and pilot testing.

A limitation is the complexity of the trial. As a result, it might be difficult to determine which specific facet of the intervention is responsible for an effect. Therefore, the specific QoL therapies patients receive at any time of measurement are recorded and will be investigated for effectiveness in subgroup analyses.

To disseminate study results, full text publications and presentations at conferences will be realised as well as the transfer of the results into the medical education of physicians and students. Furthermore, the randomised trial is an implementation study for colorectal cancer patients as well. The QoL system has already been implemented for patients with breast cancer in the area of Regensburg and is now implemented for colorectal cancer patients. The effectiveness of this modified QoL pathway in such a different patient population will be tested in this prospective randomised trial. In the long term, the QoL system is planned to be implemented for the whole region of Germany.

## Trial status

Recruitment for the trial began in January 2014 and is still in progress.

## Abbreviations

ASA score: American Society of Anesthesiologists score
CP: coordinating practitioner
QoL: quality of life.
